# Antiviral activity of Ladania067, an extract from wild black currant leaves against influenza A virus *in vitro* and *in vivo*

**DOI:** 10.3389/fmicb.2014.00171

**Published:** 2014-04-22

**Authors:** Emanuel Haasbach, Carmen Hartmayer, Alice Hettler, Alicja Sarnecka, Ulrich Wulle, Christina Ehrhardt, Stephan Ludwig, Oliver Planz

**Affiliations:** ^1^Department of Immunology, Interfaculty Institute for Cell Biology, Eberhard Karls University of TuebingenTuebingen, Germany; ^2^Institute of Molecular Virology, Center for Molecular Biology of Inflammation, University of MuensterMuenster, Germany

**Keywords:** influenza A virus, plant extract, antiviral compound, *in vivo*, prophylactic treatment

## Abstract

Influenza, a respiratory disease caused by influenza viruses, still represents a major threat to humans and several animal species. Besides vaccination, only two classes of drugs are available for antiviral treatment against this pathogen. Thus, there is a strong need for new effective antivirals against influenza viruses. Here, we tested Ladania067, an extract from the leaves of the wild black currant (*Ribes nigrum folium*) for potential antiviral activity against influenza A virus *in vitro* and *in vivo*. In the range of 0–1 mg/ml the extract showed no cytotoxic effect on three cell lines and a CC_50_ of 0.5 ± 0.3 mg/ml, on peripheral blood mononuclear cells. Furthermore, the extract did not influence the proliferative status of human lymphocytes. In contrast, Ladania067 was highly effective (EC_50_ value: 49.3 ± 1.1 ng/ml) against the human pandemic influenza virus strain A/Regensburg/D6/09 (H1N1). The extract exhibited an antiviral effect when the virus was pre-incubated prior to infection or when added directly after infection. No antiviral effect was found when infected cells were treated 2, 4, or 8 h after infection, indicating that Ladania067 blocks a very early step in the virus infection cycle. In the mouse infection model we were able to demonstrate that an intranasal application of 500 μg Ladania067 inhibits progeny virus titers in the lung up to 85% after 24 h. We conclude that the extract from the leaves of the wild black currant may be a promising source for the identification of new molecules with antiviral functions against influenza virus.

## INTRODUCTION

Influenza virus is a major plague with the potential to cause worldwide epidemics. Infection with influenza A viruses can cause mild clinical symptoms up to severe pathogenesis, an acute respiratory distress symptom and death (Peiris et al., 2010). Though there are some risk factors involved like pregnancy or chronic lung disease, the full reason for this heterogenic clinical picture remains unknown. Clinical cases of influenza A virus infection manifest in fever, cough, headache, and malaise (Louie et al., 2009; Bautista et al., 2010; Kuiken et al., 2012).

Seasonal outbreaks and occasional pandemics of influenza A viruses result in a high rate of morbidity and mortality in humans all over the world. Moreover, the emergence of the highly pathogenic avian H5N1 influenza virus, which is linked to a mortality rate of nearly 60% in infected humans, as well as the 2009 influenza pandemic (a global outbreak of a new swine-origin strain of H1N1 influenza A virus) have highlighted the public health threat and the emergence of a novel highly pathogenic pandemic influenza A virus strain (Dawood et al., 2009; Neumann et al., 2009; World Health Organization, 2012).

The annual vaccination is the first choice for protecting against influenza viruses. Unfortunately, when the prediction for the annual vaccine failed or in a pandemic situation, where in the early phase no vaccine is available, the only choices against the virus are antiviral drugs. Currently there are only two classes of antiviral drugs available that are approved and licensed worldwide against influenza A viruses for prophylactic and therapeutic treatment in humans. Neuraminidase inhibitors block the function of the viral neuraminidase and consequently virus release. Amantadine and Rimantadine are M2 ion channel blocker. Both classes of drugs have in common that virus infection of the host cell must occur before these drugs can function as antivirals. The frequency of reports describing the appearance of multidrug-resistant virus variants dramatically increased in the nearer past (van der Vries et al., 2011; Hurt et al., 2012). In this light it is disturbing that neuraminidase inhibitor resistant H1N1 influenza virus strains from the 2009 pandemic are already present (Yang et al., 2010; Meijer et al., 2011; Thorlund et al., 2011). The Centers for Disease Control and Prevention (CDC) reports that between 1 October 2008 and 30 September 2009, 1146 (99%) of 1157 seasonal influenza A (H1N1) viruses, submitted from US public health laboratories, were resistant to Oseltamivir. In addition, Sheu et al. (2010) characterized in the same period 28 seasonal influenza A (H1N1) viruses from 5 countries: the United States, Canada, China, Kenya, and Vietnam that were dually resistant to Oseltamivir and Adamantanes.

Moreover, there are very few antiviral drugs against influenza viruses available that interacts with the viral hemagglutinin in order to interfere with virus entry. In one clinical trial the function of carbosilane dendrimers as a hemagglutinin-inhibitor against human influenza viruses was investigated (Oka et al., 2008, 2009). In its actual research agenda the World Health Organization (WHO) called up to optimize the effectiveness of current and novel antiviral treatments through development of new formulations, delivery routes, or novel defense mechanisms including natural products.

There are many reports describing strong antiviral activity of traditional medicinal plants, indicating a rich source of novel molecules against influenza virus strains (Wang et al., 2006; Kitazato et al., 2007; Fiore et al., 2008). Recently, we have reported that the polyphenol rich plant extract, CYSTUS052 showed antiviral activity against influenza A viruses in cell culture and in mice (Droebner et al., 2007, 2009; Ehrhardt et al., 2007). Moreover, the antiviral activity of CYSTUS052 against seasonal influenza virus and common colds was also demonstrated in humans (Kalus et al., 2009). Notable, no drug-resistant virus variants due to CYSTUS052 treatment were found (Ehrhardt et al., 2007). In order to discover novel antiviral drugs against influenza viruses and extract from the leaves of wild black currant (*Ribes nigrum folium*) was tested. In the recent past *Ribes nigrum folium* came into the focus to gain benefit in human health. The anti-inflammatory property of this plant is due the to fact that it is very rich in tannins such as prodelphinidins and proanthocyanidins (Tits et al., 1992; Garbacki et al., 2002, 2004) as well in phenolics and antioxidants (He et al., 2010; Tabart et al., 2011). These phenolic compounds, such as flavonoids have been demonstrated to serve anti-allergenic, anti-oxidative, anti-inflammatory, anti-proliferative, anti-bacterial, and anti-viral (Gallina et al., 2011; Korkina et al., 2011; Kostyuk et al., 2011; Zandi et al., 2011; Costa et al., 2012; Ikuta et al., 2012; Pastore et al., 2012).

In the present study, we investigated the effectiveness of Ladania067 against the human influenza A pandemic strain A/Regensburg/D6/09 (H1N1, RB1) *in vitro* and *in vivo* demonstrating that Ladania067 treatment resulted in reduced virus replication without toxic effects.

## MATERIALS AND METHODS

### COMPOUNDS

Ladania067, the water-soluble extract form the leaves of wild black currant (*Ribes nigrum folium*) was supplied and originally developed by Dr. Pandalis Naturprodukte GmbH & Co. KG; Glandorf, Germany and supplied as a liquid formulation, according to the manufactories protocol. It was further lyophilized at the Interfaculty Institute for Cell Biology, Department of Immunology, Eberhard Karls University Tuebingen, Germany. The Ladania067 powder was dissolved in sterile phosphate buffered saline (PBS) or cell culture media in concentration as indicated.

Tamiflu^®^ was purchased from Roche Diagnostics (Mannheim, Germany) and dissolved in water to a working concentration of 5 mg/kg.

### VIRUS

The human pandemic influenza virus strain A/Regensburg/D6/09 (H1N1, RB1) was obtained from the Robert-Koch-Institute, Federal Institute for Public Health, Berlin, Germany. The virus was further propagated in human lung adenocarcinoma epithelial cells (A549) at the Interfaculty Institute for Cell Biology, Department of Immunology, Eberhard Karls University Tuebingen, Germany. Therefore, 1 day prior infection A549 cells were seeded in a cell culture flask to a density of approximately 40%. The day after, cells were washed with PBS and infected with the RB1 virus at a multiplicity of infection (MOI) of 5. After several days, depending on the cytopathogenic effect (CPE), supernatant was harvested. The progeny virus titer was measured by AVICEL^®^ plaque assay.

### INFLUENZA VIRUS TITRATION (AVICEL^®^ PLAQUE ASSAY)

To assess the number of infectious particles in the samples, a plaque assay using AVICEL^®^ was performed in 96-well plates as described previously (Haasbach et al., 2011). Briefly, Madin-Darby canine kidney (MDCK II)-cells grown to confluency in 96-well dishes, then they were washed with PBS and infected with serial dilutions of the supernatants in PBS/BA for 30 min at 37°C. After incubation cells were overlaid with overlay-medium [1:1, MEM-medium containing 0.2% BSA, antibiotics and 2.5% AVICEL^®^-Medium (FMC BioPolymer, Philadelphia, PA, USA)] for 24 h. Afterward, virus-infected cells were immunostained by incubating for 1 h with a monoclonal antibody specific for the influenza A virus nucleoprotein (Serotec, Duesseldorf, Germany), followed by 30 min incubation with peroxidase-labeled anti-mouse antibody (DIANOVA, Hamburg, Germany) and 10 min incubation with True Blue^TM^ peroxidase substrate (KPL, Gaithersburg, MD, USA). Stained plates were scanned on a flat bed scanner and the data were acquired by Corel DRAW 9.0 software. To define the titer of progeny virus, the foci of infected cells for every sample in each well of the 96-well plates were counted and multiplied with the dilution factor. The mean values were taken from the final number of foci in each well. The viral titers are shown as the logarithm to the base 10 of the mean values.

### MEASUREMENT OF PHARMACOLOGICAL PARAMETERS *IN VITRO*

To determine the effective concentration 50% (EC_50_) of Ladania067 against influenza A virus *in vitro* we infected 2 × 10^5^ human lung adenocarcinoma epithelial cells (A549) per well in a 24-well plate with RB1 (MOI of 0.001). After 30 min virus inoculum was discarding and the infected cells were treated with Ladania067 by adding the plant extract to the culture medium in different concentrations (0–1 mg/ml). Progeny virus titers in the supernatant of infected and treated cells were measured by plaque assay as described in 2.3. Each experiment was repeated three times independently with each comprising triplicates.

The cytotoxic concentration 50% (CC_50_) of Ladania067 was determined in A549, MDCK II and cervical cancer (HeLa) cells as well in human peripheral blood mononuclear cells (PBMCs). All cell types were seeded in 96-well plates with a density of 1.5 × 10^5^ (5 × 10^5^ PBMCs) before incubation with different Ladania067 concentrations (0–1 mg/ml) for 24 h. After incubation, cytotoxic effects were measured by a water-soluble tetrazolium salt (WST-1) assay according to the manufactures protocol (Roche Diagnostics, Mannheim, Germany). All experiments were performed in triplicates. Results evaluated by GraphPad prism 5.0 software.

### LYMPHOCYTE PROLIFERATION ASSAY AND FLOW CYTOMETRY

Peripheral blood mononuclear cells were isolated from healthy donors using Ficoll-Hypaque density gradient centrifugation (PAA Laboratories, Pasching, Austria). The cells were further incubated with RPMI 1640 medium supplemented with Penicillin/Streptomycin and autologous plasma.

Cells were transferred into 24-/ or 96-well cell culture plates and incubated with indicated concentrations of Ladania067. Pokeweed mitogen (PWM; 40 μg/ml) was used as a non-specific positive control (Biochrom, Berlin, Germany). For the proliferation assay, the stimulated cells were incubated for 6 days with Ladania067 (800, 80, 8, 0.8, 0.08 μg/ml) at 37°C and 5% CO_2_. After incubation, cells were pulsed with 0.5 μCi (2.22–3.33 TBq/mmol) of ^3^H-thymidine and further incubated for 16 h. Afterward cells were harvested and the ^3^H-thymidine incorporation was measured using the MicroBeta^2^TM Microplate Counter (Perkin Elmer, Waltham, MA, USA). For the flow cytometry analysis, stimulated, untreated or Ladania067 treated cells were incubated for 1 day at 37°C and 5% CO_2_. After incubation cells were stained with α-CD4-PE, α-CD8-PerCP, α-CD3-FITC, α-CD69-APC, α-CD19-FITC, α-CD45-PerCP, α-CD56-PE, and α-CD69-APC (Miltenyi Biotec, Bergisch Gladbach, Germany). The amount of activation in all stained cell types was measured with BD FACSCantoII^TM^ flow cytometer (Becton Dickinson, Heidelberg, Germany) using the FACS software DivaTM. The data were analyzed using the FlowJo 7.6.3 software (TreeStar, Ashland, OH, USA). All experiments were done in triplicates.

### MODE OF ACTION STUDY

The therapeutic effect of Ladania067 was determined by infection of A549 cells with RB1 (MOI of 0.001) and treatment with 100 μg/ml Ladania067 0, 2, 4, or 8 h post-infection. After 24 h incubation, progeny virus was determined in the supernatant by plaque assay. Pre-incubated cells or virus with Ladania067 were examined to show the prophylactic effect of Ladania067. A549 cells were treated 1 h prior to infection with 100 μg/ml Ladania067 or MOCK treated at 37°C and 5% CO_2_. After 1 h, Ladania067 was aspirated and cells were washed and infected with RB1 (MOI 0.001). The virus titers in supernatants were determined after 24 h. To identify the direct effect of Ladania067 against the virus, we incubated the virus with or without 100 μg/ml Ladania067 at 37°C and 5% CO_2_ for 2 h. Afterward A549 cells were infected either with the Ladania067-incubated virus or with the mock-incubated virus for 24 h. Supernatants were taken and assayed for progeny virus by plaque assay.

### VIRUS INOCULATION OF MICE

Six to eight week-old female C57BL/6 mice were obtained from Janvier (St Berthevin Cedex, France). Before intranasal inoculation with the influenza A virus strain A/Regensburg/D6/09 (H1N1, RB1), mice were anesthetized by intraperitoneal injection of 150 μl of a ketamine (Sanofi)-rompun (Bayer)-solution (equal amounts of a 2%-rompun-solution and a 10%-ketamin-solution were mixed at the rate of 1:10 with PBS).

To determine the 50% mouse lethal dose (MLD_50_), five groups of five mice were inoculated intranasally with a 10-fold serial dilution of virus. The MLD_50_ was calculated by the method of Reed and Muench (1938). The MLD_50_ of RB1 in C57BL/6 mice is 3 × 10^4^ pfu/50 μl. All animal studies were approved by the Institutional Animal Care and Use Committee of Tuebingen.

### LADANIA067- AND TAMIFLU^®^ -TREATMENT OF MICE AND VIRUS INFECTION

Thirty minutes prior to viral inoculation, mice were anesthetized by intraperitoneal injection of ketamine/rompun and treated intranasally either with 50 or 500 μg Ladania067 in 50 μl PBS. The control group received 50 μl of the solvent (PBS). Mice were infected intranasally with the 5-fold MLD_50_ of RB1. Lungs of Ladania067-treated and solvent-treated control mice were collected 24 h after infection and viral titers were determined by plaque assay.

For Tamiflu^®^-treatment C57BL/6 mice were treated 12 h prior to infection *per os* with 5 mg/kg Tamiflu^®^ in 200 μl water. The control group received 200 μl of the solvent (water). Immediately before infection with the 5-fold MLD_50_ of RB1, mice were treated again. Twelfth hours after infection mice were treated the third time. Lungs were collected 24 h after infection and viral titers were determined by plaque assay.

For the survival experiment, treatment of mice with Ladania067 was performed in an inhalation chamber (ACTIVAERO GmbH, Gemuenden/Wohra, Germany), to assure delivery directly into the lung since. Four mice were treated each in a single inhalation tube. Four of those single inhalation tubes were connected to a PARI^®^ LC SPRINT nebulizer (PARI^®^ GmbH, Starenberg, Germany). The flow rate in the inhalation chamber was set up to 8 l per min. 300 mg/6 ml PBS or buffer solution alone were given for 15 min to the chamber with a constant pressure of 2.0 bars. Treatment of mice was performed for five consecutive days twice daily every 12 h starting 6 h after infection.

For the determination of the clinical score the following disease symptoms were found and defined in mice after influenza virus infection: ruffed fur, teeth crunching, ataxia, dyspneic, conjunctivitis. If mice showed one of these symptoms they got one score; 2 symptoms = score 2; 3 and more symptoms = score 3; death = score 4. Note, the score 4 was kept throughout the 10 days observation period. Score represents the mean value of the group.

Furthermore, mice were weighted daily. When mice died, the bodyweight was kept throughout the 10 days observation period.

### STATISTICAL ANALYSIS

Error bars were given as the SEM. For the calculation of the significance of the *t*-test was performed for two different and ANOVA analysis was carried out for more than two different groups. P values of ≤0.05 are referred to as * and <0.01 as **. Analyses were performed using GraphPad Prism version 5.0 for Windows (GraphPad Software, San Diego, CA, USA) or FlowJo 7.6.3 software (TreeStar, Ashland, OH, USA).

## RESULTS

### LADANIA067 EXHIBIT NO CYTOTOXIC EFFECTS IN CELL CULTURE

**FIGURE 1:**
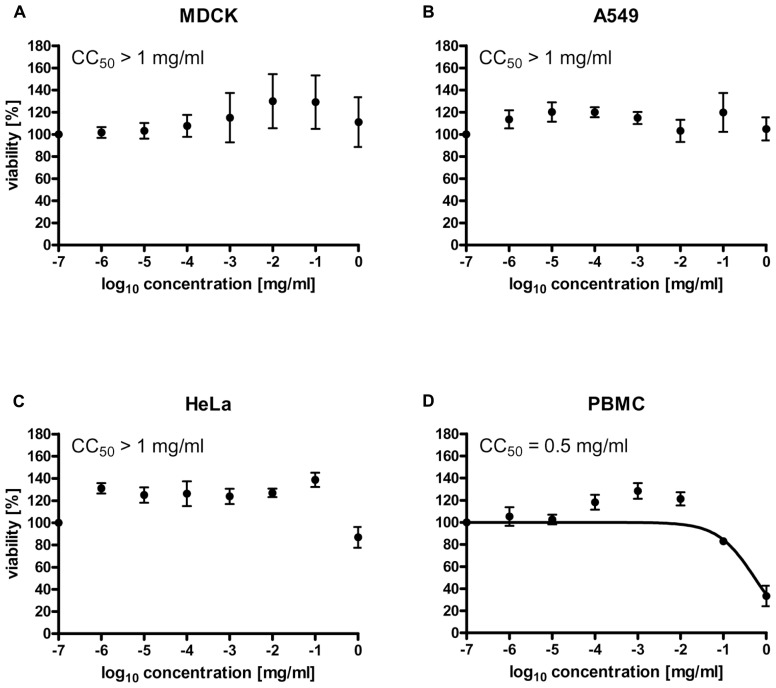
**Ladania067 treatment shows no cytotoxic effects *in vitro***. The cytotoxic concentration 50% (CC_50_) of Ladania067 was determined in MDCK **(A)**, A549 **(B)**, HeLa **(C)**, and PBMCs **(D)**. Cells were incubated with Ladania067 (0–1 mg/ml) for 24 h. After incubation, cell viability was measured by WST-1 assay regarding manufactory guidelines (*n* = 3).

To investigate whether treatment of Ladania067 would result in cytotoxic effects, the cytotoxic concentration 50% (CC_50_) of the extract was determined in four different cell types. MDCK, A549, PBMCs and HeLa cells were treated with Ladania067 in a concentration range of 0 to 1 mg/ml. After incubation period of 24 h the cell viability was measured by the WST-1 assay. The Ladania067 extract showed no cytotoxic effects against MDCK (**Figure 1A**), A549 (**Figure 1B**), or HeLa (**Figure 1C**) cells in the concentration range described. Consequently the CC_50_ value was greater than >1 mg/ml. Only with human PBMCs we could determine a CC_50_ value in the low mg range (CC_50_ = 0.5 ± 0.3 mg/ml; **Figure 1D**). These results indicate that Ladania067 either exhibits no cytotoxic effects in most cell types or only acts cytotoxic in human PBMCs in very high concentrations.

### IMMUNE CELLS ARE NOT AFFECTED BY LADANIA067 TREATMENT

Due to the fact that Ladania067 demonstrated a cytotoxic effect on human PBMCs we further wanted to scrutinize this effect and raise the question whether Ladania067 treatment would influence human lymphocyte proliferation. Therefore, lymphocytes from human whole blood were isolated and treated with different concentrations of Ladania067 (0–800 μg/ml) for 6 days following by 16 h incubation with ^3^H-thymidine. The concentrations used for treatment also included one concentration above the CC_50_ value (800 μg/ml).

**FIGURE 2:**
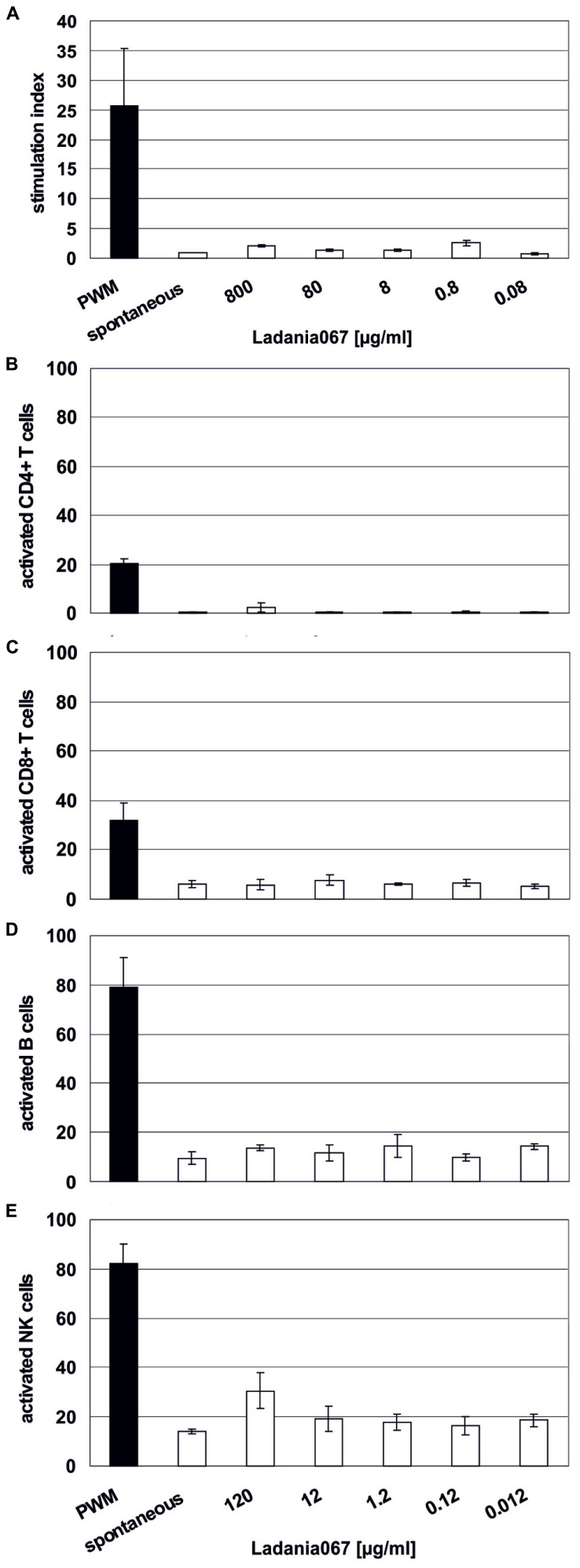
**Immune cells are not affected by Ladania067**. **(A)** ^3^H-thymidine incorporation assay was performed with fresh human isolated PBMCs. Cells were treated for 6 days with different concentrations of Ladania067 (800, 80, 8, 0.8, 0.08 μg/ml). PWM, (black bar; 40 μg/ml) was used as a non-specific positive control. After incubation cells were pulsed for 16 h with ^3^H-thymidine. Stimulation index were calculated by dividing treated cells to spontaneous release. **(B)** Flow cytometry analysis of different activated immune cell subtypes, CD4^+^ **(B)** and CD8^+^ T lymphocytes **(C)**, B cells **(D)** and NK cells **(E)** after 1 day Ladania067 treatment (120, 12, 1.2, 0.12, 0.012 μg/ml). Values are given as percent of total gated. All results represent three different donors.

First, we tested a possible influence of Ladania067 on the proliferation of human lymphocytes. Therefore, these cells were treated with different concentrations (0.08–800 μg/ml) or left untreated in order to calculate a stimulation index. As a positive control the cells were treated with PWM, a strong mitogenic stimulus for lymphocytes. While PWM readily induced lymphocyte proliferation as expected no enhanced proliferation of lymphocytes was found after Ladania067 treatment (**Figure 2A**). Interestingly, even after treatment with 800 μg/ml ^3^H-thymidine counts were comparable to the levels in cells treated with lower amounts of Ladania067 or left untreated, indicating neither a proliferative nor a toxic effect.

In addition, flow cytometry analysis was performed in order to evaluate a proliferative effect of Ladania067 against single immune cell subtypes. While PWM treatment resulted in activation of 20% of CD4^+^ T cells, almost no activated CD4^+^ T cells were found after Ladania067 treatment (**Figure 2B**). A similar picture was found for the CD8^+^ T cell and B cell population, whereas the amount of activated CD8^+^ T cells and B cells was generally increased compared to the CD4^+^ T cell population (**Figures 2C,D**). When natural killer (NK) cells were treated with 120 μg/ml of Ladania067 a slight, but not significant increase of activated NK cells was found (**Figure 2E**). Based on these results we conclude that treatment with Ladania067 neither induces cytotoxic effects nor activates lymphocytes *in vitro*.

### LADANIA067 INHIBITS INFLUENZA VIRUS REPLICATION *IN VITRO*

**FIGURE 3:**
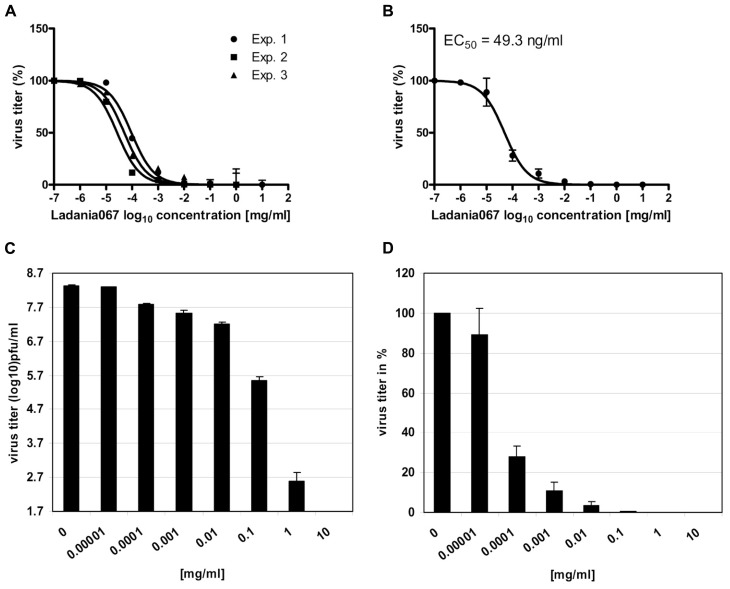
**Ladania067 reduces influenza virus replication *in vitro***. The effective concentration 50% (EC_50_) of Ladania067 was measured by treatment of influenza A virus A/Regensburg/D6/09 (H1N1, RB1) infected A549 cells. Cells were infected with RB1 (MOI 0.001) and further treated with different concentrations of Ladania067 (0–1 mg/ml) for 24 h. Progeny virus titer was determined by plaque assay. **(A)** Results of three independent experiments, **(B)** mean of three independent experiments, calculated by GraphPad Prism software. (**C**: pfu/ml) and (**D**: %) shows results in bar charts. For calculation, each experiment was performed three times independently with each comprising triplicates.

Next, we examined the antiviral properties of Ladania067 against the pandemic influenza A virus A/Regensburg/D6/09 (H1N1, RB1) in cell culture. Ladania067 was added to the A549 cells in different concentrations ranging from 0 to 10 mg/ml directly after there infection. The ability to reduce the virus titer was analyzed by measurement of progeny virus in the supernatant after 24 h of culturing. A concentration as low as 1 μg/ml of Ladania067 was sufficient to reduce virus titers by almost 90%. To determine the EC_50_ of Ladania067 against RB1 infected A549 cells the percentage of virus titer reduction of all concentrations used compared to untreated controls were analyzed using GraphPad Prism software. The experiment was performed three times with similar results (**Figure 3**). The results are presented in A and B by sigmoid function and in C and D in bar graphs. The mean values of these three experiments were plotted to calculate the EC_50_ value. As shown in **Figure 3B** the EC_50_ value of Ladania067 of RB1-infected A549 cells is 49.3 ± 1.1 ng/ml compared to a CC_50_ value for Ladania067 on A549 cells of >1 mg/ml (**Figure 1B**). The EC_50_ and the CC_50_ values allow to calculating the selective index (SI = CC_50_/EC_50_), which is the amount of drug that causes the therapeutic effect to the amount that causes cytotoxic side effects. In the present experiment the SI of Ladania067 against RBI infected A549 cells is >20.408 (Table 1). This index indicates a very broad therapeutic window of Ladania067.

**Table 1:** **Pharmacological parameters of Ladania067 *in vitro*^a^**.

	Ladania067		
	EC_50_ [ng/ml]	CC_50_ [mg/ml]	SI
RB1	49.3 ± 1.1	>1	>20.408

a Human lung adenocarcinoma epithelial cells (A549).

### LADANIA067 BLOCKS A VERY EARLY STEP IN THE VIRUS INFECTION CYCLE

**FIGURE 4:**
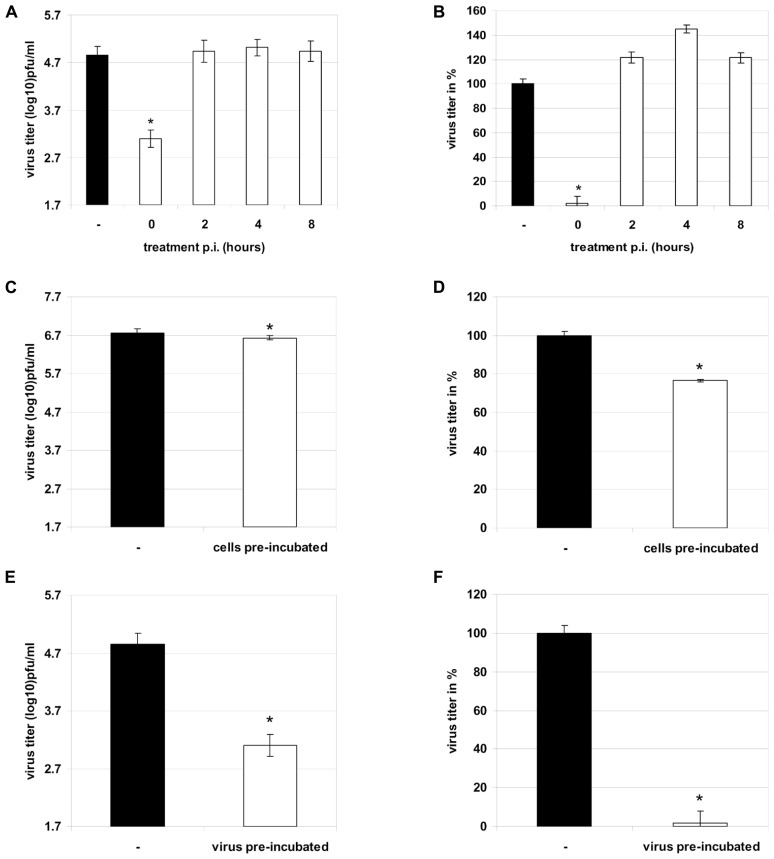
**Therapeutic treatment of RB1 infected A549 cells (MOI 0.001) 0, 2, 4, or 8 h past infection with 100 μg/ml Ladania067 or solvent for 24 h**. After incubation progeny virus titer was measured by plaque assay (**A**: in pfu/ml, **B**: in %). Treatment of A549 cells prior to infection was performed with 100 μg/ml Ladania067 or solvent for 1 h. After pre-incubation, Ladania067 was aspirated and cells were washed and infected with RB1 (MOI 0.001). Following 24 h incubation progeny virus titer was measured by plaque assay (**C**: in pfu/ml, **D**: in %). Prior to infection, virus was 2 h per-incubated with 100 μg/ml Ladania067 or solvent at 37°C and 5% CO_2_. After incubation A549 cells were either infected with Ladania067-incubated or solvent-incubated virus for 24 h. After incubation progeny virus titer was measured by plaque assay (**E**: in pfu/ml, **F**: in %). For calculation, the experiment was performed two times independently with each comprising triplicates (*p < 0.05).

Now the question arises at which stage of the virus life cycle Ladania067 inhibits influenza virus replication. First the progeny virus release of influenza A virus infected cells treated with 100 μg/ml Ladania067 was determined either direct after infection (comparable to 3.3) or 2, 4, or 8 h post-infection. A strong reduction of virus titer (1.7 ± 0.2 log_10_ pfu/ml) could only be observed when Ladania067 was added directly after infection (**Figures 4A,B** first left white bar). Ladania067 treatment at 2, 4, or 8 h p.i. revealed no antiviral effect compared to untreated control cells as shown in virus titer log_10_ pfu/ml (**Figure 4A**, second to forth white bar from the left) and in % reduction compared to control (**Figure 4B**, second to forth white bar from the left).

In another experiment the A549 cells were pre-incubated with Ladania067 at a concentration of 100 μg/ml for one hour. Subsequently, cells were infected in the absence of Ladania067. Thereafter, the supernatant of Ladania067 treated A549 cells (**Figures 4C,D** white bar) were analyzed 24 h p.i. for progeny virus release in comparison to virus infected but untreated control cells (**Figures 4C,D** black bar). Only a marginal reduction of progeny virus titer was found, when cells were pre-incubated with Ladania067. After virus pre-incubation for 2 h with or without 100 μg/ml Ladania067 at 37°C and 5% CO_2_ cells were infected either with pre-treated or non-pre-treated virus and the virus titer was determined after 24 h. A strong virus titer reduction of more than 95% could be observed when the influenza virus was pre-incubated with Ladania067 prior to infection (**Figures 4E,F** white bar), in comparison to non-pre-treated virus (**Figures 4E,F** black bar). Taken together, these experiments indicate that Ladania067 most probably interacts with the invading pathogen rather than with the host cell.

### SINGLE ADMINISTRATION OF LADANIA067 REDUCES VIRUS PRODUCTION IN THE LUNGS OF H1N1-INFECTED MICE

**FIGURE 5:**
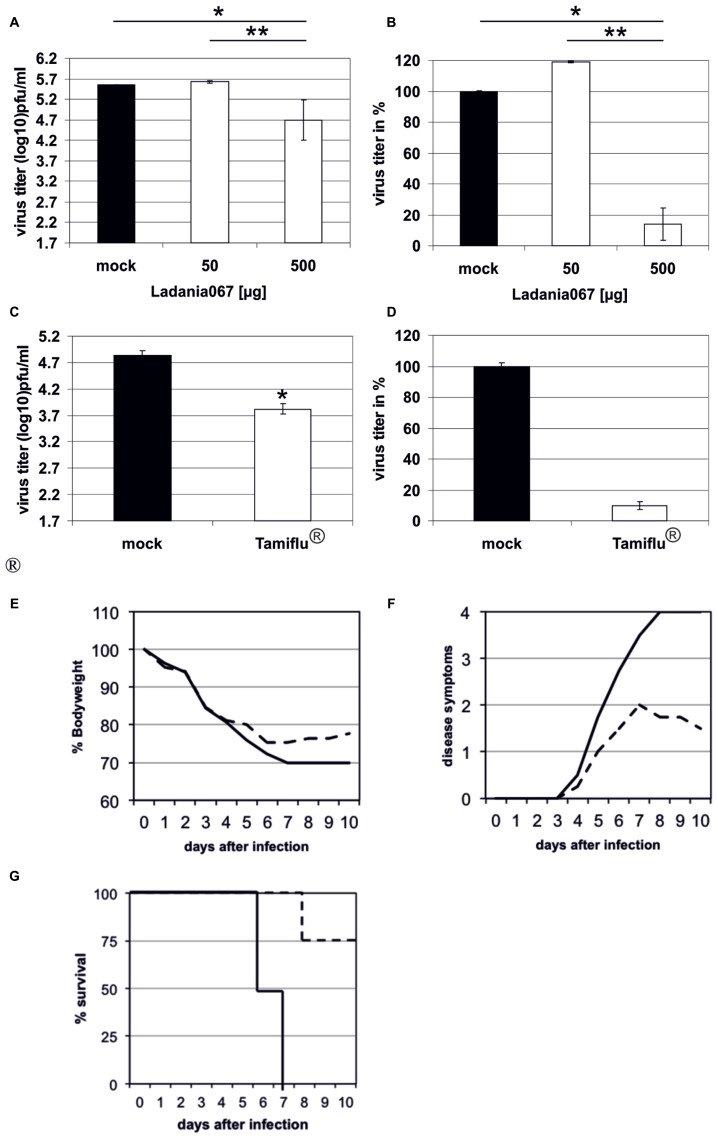
**Ladania067 administration reduces progeny virus titer in the lungs of infected mice.** C57BL/6 mice were treated 30 min prior to infection *via* the intranasal route with either 50 μg (four mice) or 500 μg (five mice) Ladania067 in 50 μl PBS. The control group (three mice) received 50 μl of the solvent (PBS). Within the anesthesia, mice were infected intranasally with the 5-fold MLD_50_ of RB1. Lungs were collected 24 h after infection and viral titers were determined by plaque assay (**A**: in pfu/ml, **B**: in %). Four C57BL/6 mice per group were treated 12 h prior to infection *per os* with 5 mg/kg Tamiflu^®^ in 200 μl water. The control group received 200 μl of the solvent (water). Immediately before infection with the 5-fold MLD_50_ of RB1, mice were treated again. Twelfth hours after infection mice were treated the third time. Lungs were collected 24 h after infection and viral titers were determined by plaque assay (**C**: in pfu/ml, **D**: in %). P values of <0.05 are referred to as * and <0.01 as **. For survival experiments **(E–G)** four C57BL/6 mice per group were either treated with 500 μg Ladania067 *via* the aerosol route twice daily for 5 days beginning with the day of infection (black solid line) or with PBS (black dotted line). Mice were weighted daily **(E)** and disease symptoms were monitored **(F)**. While untreated mice died between days 6 and 7 p.i. only one out of four from the 500 μg Ladania067 treated group died **(G)**.

After successfully demonstrating the antiviral capacity of Ladania067 in cell culture we investigated the potential of the extract to reduce influenza A virus infectivity in the mouse model. In addition we wanted to compare the antiviral effect of Ladania067 with the bona fide antiviral, Tamiflu^®^. Therefore, C57BL/6 mice were treated *via* the intranasal route with either 50 or 500 μg Ladania067 dissolved in 50 μl PBS or with 50 μl PBS (Mock) alone 30 min prior to infection. Thereafter, mice were infected with the 5-fold MLD_50_ of RB1. The lungs of Ladania067-treated and mock-treated control mice were collected 24 h p.i. and virus titers were determined by plaque assay. In the lungs of mock-treated mice a virus titer of 5.6 ± 0.01 log_10_ pfu/ml was noted. Mice treated with 500 μg Ladania067 showed a virus titer of 4.7 ± 0.49 log_10_ pfu/ml, a reduction of more than 85% compared to mock-treated mice. In contrast, mice treated with 50 μg Ladania067 showed no reduction of virus titer in the lung (**Figures 5A,B**). These data indicate that there is a dose dependent effect of Ladania067 against influenza A virus *in vivo*. Treating influenza virus infected mice with Tamiflu^®^ (**Figures 5C,D**), we found a reduction of progeny virus in the lungs of 1.0 log_10_ pfu/ml which is similar to the results of Ladania067. Therefore, in our mouse model the antiviral effect of Ladania067 is comparable with the effect of the bone fide antiviral Tamiflu^®^. Moreover, we investigated if Ladania067 would also influence the survival of RB1 infected mice. To assure deposition of the extract in the lung, Ladania067 was supplied *via* an aerosol route as performed before (Droebner et al., 2007). Four mice were infected and treated with 300 mg/6 ml Ladania067 for 15 min (**Figures 5E–G**; black dotted line) or with PBS (**Figures 5E–G**; black solid line) for 5 days twice daily starting six hour after infection. After RB1 infection both groups of mice were loosing weight, but the Ladania067 treated animals stabilized the bodyweight by day 6 p.i. (**Figure 5E**). Onset of clinical symptoms was at day 4 p.i. in both groups. While the symptoms increased in the untreated group continuously to score four (death), disease symptoms decreased in the Ladania067 treated group by day 7 p.i. (**Figure 5F**). All four untreated mice died between days 6 and 7, while only one out of four animals from the Ladania067 treated group died at day 8 p.i. (**Figure 5G**).

## DISCUSSION

There is still an urgent medical need for the development of new strategies to battle influenza virus infection. Antiviral drugs are the only source to fight the disease, when no vaccine is available, like in the early phase of a pandemic or when the prediction for the composition of the seasonal influenza vaccine fails. In 2010 the WHO suggested in their influenza research agenda the development of novel and effective treatment strategies including natural products (World Health Organization, 2010). This prompted us to investigate the antiviral activity of plant extracts against influenza virus infection.

In the present work we have chosen an water-soluble extract from the leaves of the black current (*Ribes nigrum folium*) because this plant already has a strong record in the literature for its beneficial effects for human health. *Ribes nigrum* is very rich in tannins in particular on prodelphinidins and proanthocyanidins like epigallocatechin gallate (EGCG). It is well known that these tannins have anti-inflammatory and anti-oxidant properties (Tits et al., 1992; Garbacki et al., 2002, 2004). Moreover, *Ribes nigrum* is very rich in phenolic compounds in particular flavonoids which are very well known for their anti-allergenic, anti-oxidative, anti-inflammatory, anti-proliferative, and antiviral properties (Gallina et al., 2011; Korkina et al., 2011; Kostyuk et al., 2011; Zandi et al., 2011; Costa et al., 2012; Pastore et al., 2012). In addition, EGCG shows anti-influenza virus activity (Song et al., 2005; Kim et al., 2013).

In previous studies with a polyphenol-rich extract from the plant *Cistus incanus* CYSTUS052, we were able to demonstrate a pronounced antiviral effect against influenza A virus *in vitro* and *in vivo.* The underlining mechanism is the blocking of virus entry. This blockade is most due to an unspecific anti-adhesion effect on the pathogen since a specific interaction with cellular receptors in particular with sialic acid receptors could not be demonstrated (Droebner et al., 2007; Ehrhardt et al., 2007).

In the present work we were able to demonstrate that the extract from *Ribes nigrum folium* (Ladania067) has strong antiviral activity against influenza virus. The EC_50_ value of Ladania067 in RB1-infected A549 cells was 49.3 ± 1.1 ng/ml. This very potent activity leads to the assumption that the secondary plant metabolites responsible for the antiviral activity must be present in high concentrations. In contrast, the CC_50_ value on primary human PBMCs was 0.5 ± 0.3 mg/ml. No toxic effects were found in all cell lines tested, when concentrations of up to 1 mg/ml were used. Moreover, neither a stimulatory nor an inhibitory influence on primary human lymphocytes could be measured. From these data one can conclude that Ladania067 has a very broad SI. As demonstrated here, on human A549 cells the SI value is >20.408. The results also demonstrated that the antiviral function was only present, when the extract was added to the cell culture system prior to infection. Treatment that started two hours after infection was not successful in protecting the cells. In contrast, when the virus was pre-incubated with Ladania067 a strong reduction of virus titer was present. From these results we conclude that Ladania067 has virucidal properties but might also blocks the virus replication in a very early step of infection; most probably by a similar anti-adhesive manner like CYSTUS052 (Droebner et al., 2007).

It is well known that plant extracts can efficiently inhibit viral attachment and penetration. In the case of herpes simplex virus type-1 (HSV-1) there were different plant extracts, *Myrothamnus flabellifolia*, *Rumex acetosa* L., or *Azardirachta indica*, which act as a blocking agent of viral adsorption and/or penetration (Tiwari et al., 2010; Gescher et al., 2011). Nevertheless, secondary plant metabolites such as theaflavin, pentagalloylglucose, or glycyrrhizin can also function in a direct manner. Theaflavin is a compound that is able to inhibit the influenza virus replication *via* interference with the haemagglutinin synthesis. Moreover, it can also decrease the expression level of the inflammatory cytokine IL-6 during viral infection (Zu et al., 2012). Pentagalloylglucose and glycyrrhizin also show anti-influenza virus properties which are mediated by reduced activation of NFκB, JNK, and p38 or an interaction with the cell membrane which most likely results in reduced endocytotic activity and hence reduced virus uptake (Wolkerstorfer et al., 2009; Liu et al., 2011; Michaelis et al., 2011). In another study it was shown that EGCG, epicatechin gallate (ECG), and epigallocatechin (EGC) demonstrate antiviral effect against influenza A and B viruses. This is mediated by specific interaction with the hemagglutinin and by altering the physical properties of viral membrane (Song et al., 2005). Resveratrol, another polyphenol found in grapes strongly inhibited the replication of influenza virus in cell culture by blockade of the nuclear cytoplasmic translocation of the viral ribonucleoprotein complex, by reducing the expression of late viral proteins and by inhibition of protein kinase C (PKC) activity and PKC-dependent pathways (Palamara et al., 2005).

Due to the data obtained from the influence of Ladania067 on lymphocyte proliferation or activation we can exclude such direct effect. In particular alterations on cytokine expression would have an effect on the tested human PBMCs. This was not the case at least for the activation or proliferation of CD4^+^ and CD8^+^ T lymphocytes, NK or B cells (**Figure 2**). Therefore, we hypothesize that the full plant extract Ladania067 rather interacts with the pathogen to block virus attachment to the target cell.

In order to validate our *in vitro* findings animal experiments were performed in the mouse, where we were able to demonstrate that treatment with the extract of *Ribes nigrum folium* was successful in reducing the amount of influenza virus in the lung. We decided to treat mice *via* the intranasal route, since this is the natural route of influenza virus infection and the extract was directly delivered to the place of infection and were able to show that Ladania067 exhibits similar antiviral effects *in vivo* as Tamiflu^®^. The results indicate that the findings from our *in vitro* experiments can be transferred to the situation *in vivo*. Treatment of mice started prior to infection and moreover, best antiviral effects *in vitro* were found when treatment was started before infection. Furthermore, survival experiments were performed indicating protection from death after Ladania067 treatment. From these data we conclude a possible prophylactic function of this plant extract that is non-toxic in cell culture and also a therapeutic function in early stages of the disease.

In summary, treatment with the plant extract Ladania067 from the leaves of the black currant, leads to a virus reduction *in vitro* and in the mouse model. At the present stage we conclude that Ladania067 could be a very promising extract for the development of a new, safe and efficient antiviral compound, based on herbal products. It can act as a novel defense strategy against influenza virus infection, even tough the effect is mostly virucidal. Thus the compound might be used prophylactically but also in an early therapeutic setup to prevent the virus spead. In addition to the virucidal functions our findings support the work of Ehrhardt et al. (2013), which showed that the protective effect of Ladania067 appears to be mainly due to interference with virus internalization. Furthermore, this work demonstrated antiviral activity of Ladania067 against two other viruses A/FPV/Bratislava/79 (H7N7) and A/Puerto Rico/8/34 (H1N1). Thus, at this stage of the development it is full in line with the recommendation of the WHO influenza research agenda.

This study is another example that plant extracts are able to inhibit influenza A virus infection *in vitro* and *in vivo.* From these findings one might take two further research directions. One would be to further characterize and identify the compounds with antiviral properties in the extract. The antiviral activity of EGCG was already described. Nevertheless it is of great importance to answer the question whether only one compound, or several closely related compounds, or a composite activity of several different molecules are responsible for the observed antiviral activity and whether is activity is selective for influenza viruses? Moreover, since the extract is already used in human nutrition and shows no adverse effect a clinical pilot study to would give further information on the potency of the full extract to protect against influenza virus infection.

## Conflict of Interest Statement

The authors declare that the research was conducted in the absence of any commercial or financial relationships that could be construed as a potential conflict of interest.
